# Helping palliative care healthcare professionals get the most out of mentoring in a low-income country: a qualitative study

**DOI:** 10.1186/s12904-016-0164-x

**Published:** 2016-11-04

**Authors:** J. L. Whitehurst, J. Rowlands

**Affiliations:** 1 Guy’s and St Thomas’ NHS Foundation Trust, London, UK; 2 Aneurin Bevan University Health Board, Newport, UK

**Keywords:** Palliative care, Low-income country, International health volunteering, Being a mentor, Mentorship, Monitoring and evaluation, Global heath partnerships

## Abstract

**Background:**

Being a mentor in any setting brings challenges in addition to recognised benefits. Working in a low-income country confers specific challenges including logistical and communication issues. The need to adequately support UK-based international health volunteers prior to, during and after their trip is recognised at government level. Whilst the need to support mentors is recognised little is known about their support needs. This study aims to explore the lived experience of mentorship in a low-income country and gain insight into mentors’ support and information needs and the barriers and facilitators to mentoring.

**Methods:**

Purposive sampling was used to recruit UK-employed, palliative care clinicians: four consultants, two specialty trainees, and two nurses, who were mentors with an international palliative care project. Semi-structured telephone interviews were recorded and analysed using interpretive phenomenological analysis.

**Results:**

Participants became mentors to help others. Uncertainty about their achievements constituted a significant challenge. This study highlights the need to prepare mentors before their in-country visits by exploring motivation, describing the reality of international volunteering and ensuring realistic expectations. Post-trip debriefing is important for reducing uncertainty around trip outcomes and maximising transferable impacts. Challenges to mentoring were logistical, related to the concept of mentorship and cultural. Facilitators included shared passion, mentor credibility and serendipity.

**Conclusion:**

Awareness of the support needs of mentors and the facilitators and challenges to mentoring can improve mentor preparation and support. This may minimise potential negative emotional impact of being a mentor, maximise positive personal and professional impacts and improve in-country project impact.

**Electronic supplementary material:**

The online version of this article (doi:10.1186/s12904-016-0164-x) contains supplementary material, which is available to authorized users.

## Background

Mentoring is a developmental relationship aiming to recognise and support the mentees potential for personal and professional growth, whilst creating or expanding opportunities for success [1]. The mentor role is multidimensional with numerous facets including: teacher, facilitator, networker, coach, counsellor, confidant, nurturer, critical friend, and role model [1–4]. Mentorship can occur on a one-to-one basis, between an individual and a group, between peers and within a multiple-mentor model which recognises the need for mentors with complementary skills at different times [3, 5].

Being a mentor brings recognised personal and professional benefits and can benefit institutions [1, 2, 6–9] However the mentor role in any setting can also be disappointing [1, 5]. Time constraints, stress and frustration have all been identified as major challenges to mentoring [7, 10] Mentors can find ‘being a mentor’ permeates their entire working role, increasing fatgue [10]. Mentors can struggle to manage competing mentoring and clinical demands and feel unprepared and overwhelmed by their role [11]. Personality clashes; competition; misunderstanding of goals, roles and boundaries; and discordant roles, including that of mentor and assessor, can all render a mentor relationship unbeneficial [2, 3, 5, 12].

It is likely that the roles of international mentor and international health volunteer overlap. Published studies exist exploring the impact of international health volunteering, including a 2015 qualitative study exploring the personal value of being involved in a project supporting a palliative care degree programme in Uganda [13]. These studies cite a range of personal and professional benefits to international volunteering and mentorship encompassing teaching, management, leadership clinical skills and personal growth [13–16]. Additional challenges linked to working as an international volunteer have also been indentified with further insight available from an unpublished survey of 41 international PC volunteers. (Leng, personal communication) Challenges included: cultural and language differences; communication, logistical and resource issues; personal financial and family considerations; lack of work place support; finding an appropriate role and professional relevance [14, 15], (Leng, personal communication)

The need to adequately support international volunteers prior to, during and after their trip, has been stated by the Department of Health & Department for International Development [15].

During a study exploring the personal, professional and institutional impact of being a mentor to a palliative care (PC) team in a low-income country it became apparent that the lived experience and impacts of mentorship were intimately linked to the experience of being a mentor within a specific project – the level of preparation and support that this provided. This resulting paper is concerned with the support and information needs of mentors and identifies factors that can maximise the success of mentoring projects.

## Methods

This study was undertaken by J.W a specialty registrar for her MSc in Palliative Medicine at Cardiff University. J.W performed all the interviews. J.R acted as supervisor. Reporting is in line with the Consolidated Criteria for Reporting Qualitative Research Additional file 1.

### Participants

Purposive sampling was used to recruit current mentors from an international palliative care initiative aiming to support the development of an integrated model of PC across four African countries. All mentors had visited a hospital-based PC team either as an individual or in a group of up to four mentors for a period of 2 weeks.

Inclusion criteria: Currently working as a PC doctor or nurse in the UK, in-country visit completed 6–12 months prior to interview. Exclusion criteria: Retired, non-UK based and non-specialist PC mentors (e.g. General Practitioners).

Mentors received an email containing a participant information leaflet (PIL) from the initiative’s mentor coordinator requesting permission to be contacted by J.W. The PIL contained the study’s aim, design and academic purpose. J.W also approached mentors at an initiative event following a spoken address and distributed PILs before gaining consent.

### Data collection

Semi-structured telephone interviews occurred from home and lasted 47 minutes on average. The interview schedule was informed by: potential rapport, communication and interactional issues affecting telephone interviews [17–19]; the research question; relevant literature; and personal experience of J.W - 15 months working in PC in a low-income country and mentor in the same initiative. It was revised following the pilot. Verbal cues and active listening techniques compensated for loss of visual cues [17–19]

### Data analysis

Interviews were digitally recorded and transcribed verbatim. All transcripts were read and analysed by J.W. J.R reviewed the pilot and a sample of subsequent transcripts to ensure accuracy and consistency.

Data analysis followed interpretive phenomenological analysis principles [20]. Transcripts were read alongside the digital recording to check accuracy. Three subsequent readings aimed to allow immersion; explore descriptive, linguistic and conceptual components; identify emerging themes; check for overlooked themes. A coded master-grid of emergent themes was compiled and themes from each interview compared looking for commonality and divergence. Themes were identified through the participants’ narratives and through the interpretation of the narratives by the interviewer. All themes stemmed from the text. Related themes were clustered to produce super-ordinate and sub themes. Participants were provided with a summary of the research findings after completion.

## Results

Eight out of nine identified candidates agreed to participate; the remaining candidate did not answer introductory emails. Final analysis includes seven participants; the pilot interviewee did not answer emails seeking revised consent. Participants included six PC clinicians: two specialty trainees; four consultants, ranging from newly appointed to approaching retirement; one PC nurse.

Mentors’ experience of mentoring and global PC varied considerably. Four had received some training in mentorship prior to involvement in the project but only two had been involved in a formal mentoring programme – one as mentor and one as mentee. Other mentors had informal experience of mentorship.

All mentors had some prior experience of international PC but this ranged from short duration (1 to 8 week) teaching visits in a variety of low-income countries in four cases, to a period of working in a low-income country for greater than 1 year (maximum 8 years) in three cases. Participants are referred to by a numerical identifier and individual demographic data is not included to reduce the ability to attribute quotes to an identifiable individual.

Three key areas emerged exploring what helps mentoring be effective: supporting the mentor, challenges to mentoring and facilitators to mentoring. Table 1 summarises the identified super-ordinate and sub themes.

**Table 1:** Emergent themes

Super-ordinate theme	Sub themes
A. Supporting the mentor	A1 Exploring motivation and assessing competence
	A2 Pre-trip preparation and previous experience
	A3 The importance of debriefing
	A4 Building on trip impact after return to the UK
B. Challenges to mentoring	B1 Logistics – communication, time-constraints and availability
	B2 Finding a role
	B3 Cultural – personal and institutional
C. Facilitators to mentoring	C1 Shared passion
	C2 Credibility of the mentors
	C3 Serendipity

### Supporting the mentor

Despite common themes the lived experience of each mentor was unique. This was influenced by the individual circumstances of the mentor visit, mentors visited six hospitals in three countries, but also by how prepared mentors felt and by how much feedback they got after their visit. All mentors came from the same project and experienced similar pre-trip preparation and post trip communication yet they perceived these differently. This suggests there are mentor specific factors that also affect their lived experience and influence their support needs.

#### A1: Exploring motivation and assessing competence

A humanitarian drive - to ‘do good’ and to be useful, was common across participants. This incorporated a potential benefit to self through feelings of fulfillment. Other drivers differed between participants and were related to their personal situation: Four mentors cited the opportunity to return to Africa having previously worked, taught or lived there; one recognised the potential for professional development; another, who was nearing retirement, valued the opportunity to continue to contribute to the development of PC. Project design also attracted participants: one mentor cited the emphasis on mentoring rather than teaching; another cited its duration, as they would not have been able to undertake longer term volunteering; one felt the short duration of mentor visits to be of professional interest, questioning if it would be effective. Some mentors described more than one driver and the hierarchy of drivers differed between mentors.

*“A desire to spread information and knowledge – also selfish, because I get sickened by our profligacy and waste and self-satisfiedness. I’m talking about me probably as well as institutions. And I go because maybe I have this misperception that I (laughs) do good, feel good and look good. But actually I do it because I love it.” P7*

*“I guess there were personal and professional reasons why I felt that it would be helpful. [] I thought it would be a useful experience for me to help develop the leadership in service development skills, which to me as a clinician and a doctor in the UK was important. [] And at the same time I guess it was almost a way of being able to combine work with a hobby [] in terms of, you know, just enjoying being in Africa, forming friendships and relationships with the African people.” P4*

It appears that the greater the expectation mentors placed on themselves to succeed as mentors and humanitarians, the greater the depth and persistence of negative emotions such as disappointment or uncertainty if expectations were not met. Despite being unable to form a mentor relationship during their visit one mentor viewed their contribution more generously than other mentors who had formed mentoring relationships. This may be because a main driver for this mentor was professional interest and they could also recognise success in delivering large group teaching during their visit.

Having chosen to become mentors some described the need to check their suitability. This was usually an internal process of assessment but one mentor voiced a wish for external verification. Two mentors expressed guilt about the financial cost of in-country visits. One mentor felt using local mentors and resources could have been a more cost-effective approach and felt discomfort when patients asked them for money. Participants who described fulfilling their drivers and aims questioned their legitimacy less than those who did not.

*“Well, I hadn’t really sort of thought about whether I was equipped as a mentor before I went. I mean our roles [describes clinical role] in that job you’re always attuned to the needs of other people and what’s needed and how you’re going to get from A to B and how you’re going to support them to do that. So I guess it’s almost inherent in your role.” P6*

*“It makes me feel uncomfortable. I feel if I’m going to go and do something like that I needed to – people needed to know who I was, where I’d come from, what my experience was, whether I’d be able to do anything useful. I think all of us should have gone through a more stringent appraisal.” P7*

#### A2: Pre-trip preparation and previous experience

Mentors agreed that preparing for their mentor visit was difficult and that this was not unexpected. Mentors with more low-income country experience appeared to feel more comfortable taking on the unknown and more able to rationalise and accept logistical issues than those with less experience. This was due to a combination of foreknowledge about what low-income country PC looked like and awareness that logistical issues are to be expected. For those with less experience acknowledging that it is normal to feel somewhat unprepared may be helpful. Sharing stories from returned mentors may help them feel more prepared as they learn from others experience. Inexperienced mentors may need more direction about what activities to undertake when in-country and how to initiate them.

*“I think we were given a reasonable amount of information, and I think we sort of acknowledged as a group that we probably had enough and as much as we might expect to get from a resource poor country really. I think we would always have liked a bit more in terms of what was going on in the hospital at the time but, yeah, we acknowledged that we probably got as much as could be expected about the team.” P4*

*“I’ve done a lot of working abroad in palliative care teaching, so I’m used to the unexpected, I’m used to the hierarchy, I’m used to things getting cancelled and things changing, “P2*

*“So did it prepare me? And then the weekend that I went on before, yes, I mean it was definitely better than not having gone onto it at all. But I think because it was all so new and I hadn’t been out there or anything that, you know what I mean, I didn’t really know what I was expecting.” P5*

*“I would have found it quite a very difficult experience in two weeks if I hadn’t been to Africa before. It would have been overwhelming actually; I would have no idea what to do.” P6*

#### A3: The importance of debriefing

A key challenge, described by most mentors was uncertainty regarding the value of their visit. The extent to which uncertainty troubled the mentors varied and may relate to pre-existing patterns of seeking validation. Only one mentor had received contact from a mentee in the month preceding interview. A perceived lack of feedback from the project compounded uncertainty for those seeking external legitimacy but also introduced uncertainty for mentors who had otherwise been content with their contribution.

*“I mean it was a really enjoyable experience. And er I guess perhaps it sort of would be nice to know what happens next, and do they want more involvement? You know, because a lot of time, effort, money has gone into the project, and what have been the results? Um has [place name] got a palliative care team” P6*

Mentors found reassurance when other mentors shared their challenges; through discussions within mentor teams and/or through reading mentor visit reports and attending project events. Comparing experiences was not always reassuring, particularly if another mentor was perceived to have been more successful. Below, two participants give contradictory explanations for some of the challenges they faced linking mentee hospital size to the scale of their impact. This exposes the personal rather than universal truth of experiences and the explanations attached to them.

*“So I felt – hmm. And have I done anything useful? Um because I read people like [name] report and I think, “Oh, I didn’t get any of that. I didn’t achieve any of that.” I think, well, it was a very different situation that [name] went to. It was a big teaching hospital.” P7*

*“Talking to the other mentors and reading their blogs, reports and things I think they've often had a much more positive experience and I think that's because they go to small hospitals where is easier to have a large impact in a short amount of time.” P1*

Even within their own narratives some participants gave contradictory assessments of the relative success of their visit, post-visit communications and how they felt about this. This exposes the steps taken to make sense of their experience in isolation and is shown in the following two extracts from participant five.

*“But it did feel like there were certain things everyone [mentors and mentees] agreed would be good developments to have, and yet they don’t all seem to have kind of gone ahead. And so I think you do – you are left slightly with the sense of helplessness and not quite knowing what’s going to happen. But I guess some of that is that you have to learn to cope with it really, and you can’t make that happen and you can’t be in control of it [] and so that’s slightly uneasy, unsettled, almost like unfinished kind of position really.” P5*

*“I think having someone visit and just have the opportunity to talk about what they’re up to and just show your experience, I think it would be great. I think, if it was me and someone came and just was interested really and was positive about it, I think that would be great. I don’t think the fact that there’s no ongoing mentorship there by the UK team is a problem particularly.” P5*

Only one mentor did not question whether they had been useful as a mentor. They had received external endorsement by being invited back to the mentee hospital by its PC team and the national PC association. This supports the assertion that external feedback is integral to feeling valued and useful as a mentor.

#### A4 Building on trip impact following return to the UK

Mentors commented on how supported they felt by their UK colleagues and employers following their mentor visit. The professional and institutional impacts of international mentoring may be affected by colleague and employer perceptions of its relevance to their own service. Awareness and acknowledgement of the potential benefits and challenges of mentorship may help translate transferable skills into actions.

In this study, one mentor described feeling undervalued by their employing organisation when the additional skills they had developed through international health volunteering went unrecognised.

*“Yes, yeah and it reaffirms you again somehow. Coming back to the system here, you just get lost. You know, often you don’t feel valued or anything: you’re just a pair of hands that does what it says on the box.” P6*

Another mentor’s employers had taken steps to enable international work to continue, highlighting the appreciation employers had for the mentor and their skills but also the impact of fatigue that can result from undertaking international work during annual leave from UK work.

*“I used to take it [international volunteering trips] in the holidays and then he [boss] got fed up when he thought I got overtired and I needed a holiday, so he’s given me a month’s unpaid leave, which is wonderful” P7*

Mentors may have benefitted from discussing strategies to increase employer and colleague awareness regarding visit relevance with fellow mentors or the project team.

### Challenges to mentoring

#### B1: Logistics: communication, time-constraints and availability

Some participants felt that improved logistics would have increased mentoring success. One mentee hospital was not expecting the mentors on the day they arrived and in another hospital potential mentees were enrolled on a course. One mentor described changing countries at short notice, which they found unsettling.

The short duration of the mentor visit meant mentors had to develop relationships and set goals quickly. One mentor felt this limited their ability to mentor.

*“I think the time was really short because we were engaged in a training program the whole time the first week and in the second week they were all doing other things so actually forming even a personal friendship is quite difficult in a limited time period so that didn’t help as well.” P1*

The majority of mentees in the project provided a palliative care service in addition to other clinical responsibilities and as such were not always available to mentor. One participant felt the frustration of competing clinical responsibilities was shared between mentors and mentees.

*“So there wasn’t anyone employed specifically for part of the palliative care team, so that was one of the frustrations sometimes, I think, for them, and for us, because they had to go off and do their other jobs”.P3*

#### B2: Finding a role

It was not always clear who should be being mentored or if mentees understood the concept of mentorship. Some mentors had to negotiate whom they were mentoring. This suggests there may be value in preparing mentees adequately before mentorship can take place. Discordant perception of mentoring between mentor and mentee is likely to impact effectiveness [8].

*“So it was new, the word [mentoring] was new to them, so it became very confused [] and then the question was who were we mentoring then? And we were directed to mentor the students whereas it might have been better if we had mentored those who were mentoring, if you see what I mean.” P2*

Four of the PC teams or their hospitals were also receiving mentorship or input through pre-existing projects that may have affected their motivation to engage with mentors.

#### B3: Culture

Institutional Culture: All mentors met with hospital management in an advocacy role supporting access to resources, personnel and clinical time with variable levels of success. Whilst some managers shared the vision for PC other mentors saw a lack of engagement as a barrier to developing the service.

*“And perhaps one of the most important and the thing that provided the biggest obstacles was trying to get the senior administration on-board with needing a palliative care service. Because their attitude was, “Well it’s great we’ve got these people trained, thank you very much, but, you know, we’re under-resourced, these people are going back to the jobs they came from and we’re not planning to put them into palliative care. So they can do that above and beyond their - palliative care - er their normal role.” P6*

Personal culture: Language and cultural barriers to mentoring were also identified. One mentor found senior doctors and many nurses to be more competent in French than the language they used when mentoring, English. Whilst several mentors described non-specific cultural differences, participant two reflected on the difference in the culture around teaching and learning and communication.

*“Um it felt quite tricky because their um – the culture as we could perceive it was: I am the teacher; you are the pupil. And mentoring is much more free flowing, it’s relational, it’s to and fro, it’s challenging the mentor as well. It was quite difficult to get away from: I am the teacher; you are the pupil situation.” P2*

*“Email and long distance is not something that they’re comfortable with. I was advised by [mentor coordinator], for instance, if you send an email to two people neither will answer it, (laughs) which seemed to be true.”P2*

Lack of uptake of mentoring by email is likely to have been influenced by but not restricted to culture. Some mentors also acknowledged the technological and resource barriers citing difficulties accessing a computer and the Internet for some mentees. It has been suggested that mentorship by email requires training for both sides on how to phrase and respond to complex queries [21].

### Facilitators to mentoring

#### C1: Shared passion

The vision and enthusiasm of many mentees allowed mentoring activities to occur as they sought to prioritise mentoring against competing responsibilities. This increased the respect mentors had for their mentees, which in itself may enable the reciprocal aspect of mentoring.

*“Yes, I guess probably in the UK you don’t find - perhaps I’m just old – but you don’t find people with such passion. Um and perhaps because there’s a lot of pioneering work that needs to be done in Africa, you can get that passion, “Yes, this is something that needs to happen.” It’s, “How can I make it happen?” you know, just that raw energy to try and improve things.” P6*

There was also acknowledgement that mentees were more likely to take up the support offered by a mentor if it coincided with their own learning and development needs. Mentors did not appear to find this unusual or unexpected suggesting they had experienced similar with colleagues in the UK.

*“I think the two who we spent the most time with were the two doing the palliative care degree and diploma, I guess because it felt that it was a bit clearer to have some objectives with them because they had clear goals in terms of their personal palliative care development.” P4*

#### C2: Credibility of the mentors

How credible mentors perceived themselves to be and were perceived to be by others was felt by some to influence the success of mentoring. Previous experience of PC in a low-income setting was important for credibility but was not the only factor: age, sex and clinical background were also cited.

*“But knowing much more about what is available on the ground rather than kind of going out and basing your knowledge about – from the UK really I guess. Because it is so completely different, the resources that are available and the medications that we might use over there are different.” P3*

Some mentors travelled individually and others in teams. Those who mentored in teams reflected on the benefit of having a skill mix within the team. Conversely two mentors suggested that an individual or small mentor team would be most cost-effective. This again highlights personal rather than universal truths.

#### C3 Serendipity

Many participants described using opportunistic mentoring to maximise impact. Reaching outside the immediate mentee group to other HCPs and hospital management and the timely coincidence of a mentors visit were both described as contributing to success.

*“And if he’d come back [from palliative care training] to just the hospital and these administration staff who were just wanting to send him back to outpatients, it would be very easy to get despondent and not know where to go. But because a mentor was there they could link in with that and it gave it some impetus and importance and, you know, felt like somebody cared and wanted this to happen, and a way to channel his enthusiasm.” P6*

## Discussion

This study provides insight into the support and information needs of mentors and the challenges and facilitators to mentoring. Increased knowledge regarding the reasons people become mentors, the challenges they face and their information and support preferences could help organisations that oversee mentor projects select, prepare and support mentors. This in turn may impact on both the success of projects that use mentors in low-income countries and on the reputation of such projects.

Ultimately mentors wanted to fulfil their humanitarian drive but they also carried self-doubt and needed to know that they had been useful. The ability to fulfil drivers influenced the personal emotional impact of being a mentor. The un-met need for external endorsement, to be told they had done a good job, was a driver for ongoing uncertainty and disquiet. Seeking to fulfill a variety of expectations through the same experience will have different perceived outcomes across mentors. Exploring the motivation and expectations of mentors at the preparation stage would allow a discussion of how realistic their expectations are. Feedback to mentors both on the outcome of their own efforts and if it applies, the project as a whole may help reduce uncertainty and increase sense of worth.

This study also provides insight into the facilitators and barriers to mentoring. Shared challenges identified in this and other studies include communication and logistical issues, the lack of resources and work place support [14, 15], (Leng unpublished) – most mentors in this study took annual leave to allow them to visit their mentees. Sharing challenges between mentors helped to normalise them and protect from negative emotional responses.

It was not possible to identify who the ideal mentor is because response to the mentoring experience is informed by a complex interaction of personal and professional factors. Previous experience working in a low-income country and the duration and depth (e.g. working rather than teaching) of this experience was protective - helping mentors feel prepared and able to cope with challenge in most cases. This has also been described by Busse et al [14] who cited prior experience along with multiple prior trips and a desire for career enhancement, as being associated with a positive impact on professional development.

Restricting mentors to an experienced group would reduce the selection-pool and prevent others benefitting from the opportunity. Pairing inexperienced with experienced mentors and acknowledging the different level of support mentors might need to reach a similar level of preparation may overcome this. Awareness of potential impacts of projects on mentors may also improve monitoring and evaluation methods [14]. Mentors in this study were all palliative care specialists. However the identified support needs are likely to be relevant to generalist palliative care providers involved in international volunteering and mentoring projects, for example General Practitioners.

This study also highlights the need to prepare mentees and their institutions to receive mentors. A shared project definition of mentorship capturing its multidimensional nature and including the mentorship of individuals, teams and institutions could aid this.

### Limitations of study

This study analysed data from only seven participants due to the limitations of an MSc project and the small number of potential participants. Challenges and the resulting support needs identified concur with those in other studies concerned with international health volunteer work [14, 15], (Leng, personal communication) and the final evaluation report of the wider initiative [22]. All mentors were drawn from a single initiative and initiative factors influenced their experience. Despite this, mentors possessed a range of mentoring and low-income country previous experience and visited a number of different countries and institutions increasing the study generalisability. Mentors were interviewed 6 to 12 months following their visit and recall bias may affect the validity of their responses. Interviews immediately post in-country visit may have given different results as the post-visit mentoring challenges and uncertainty of outcome evolved over time. All participants had met J.W as a fellow mentor and one participant was on the same mentor team. Responses may have been influenced by loyalty to the initiative, a desire to help the first author and concerns that attributable comments would be fed back to the initiative. Dual roles of researcher and mentor had potential to bias analysis, maintaining awareness and analysis review by J.R reduced this risk.

## Conclusion

Awareness of the support needs of PC mentors volunteering in a low-income country and the facilitators and challenges to mentoring can improve the preparation and support of mentors. This in turn may minimise the potential negative emotional impact of being a mentor and in doing so maximise positive personal and professional impacts and improve initiative impact, effectiveness and reputation.

## Additional file

Additional file 1: Consolidated criteria for reporting qualitative studies (COREQ): 32-item checklist. (DOCX 19 kb)

## Abbreviations

HCP: Health Care Professional
J.R: Julie Rowlands, MSc supervisor
J.W: Jane Whitehurst, first author
PC: Palliative Care
UK: United Kingdom
